# Non-vitamin K antagonist oral anticoagulants versus warfarin in atrial fibrillation patients with heart failure and preserved, mildly reduced, and reduced ejection fraction: A systemic review and meta-analysis

**DOI:** 10.3389/fcvm.2022.949726

**Published:** 2022-07-29

**Authors:** Kaisaier Wulamiding, Zixuan Xu, Yili Chen, Jiangui He, Zexuan Wu

**Affiliations:** ^1^Department of Cardiology, The First Affiliated Hospital of Sun Yat-sen University, Guangzhou, China; ^2^NHC Key Laboratory of Assisted Circulation, Sun Yat-sen University, Guangzhou, China; ^3^Department of Emergency, The Third Affiliated Hospital of Sun Yat-sen University, Guangzhou, China

**Keywords:** atrial fibrillation, anticoagulants, heart failure, warfarin, meta

## Abstract

**Background:**

Patient prevalence of atrial fibrillation (AF) and heart failure (HF) is increasing, and anticoagulation for patients from heterogeneous backgrounds with both conditions remains controversial. In this meta-analysis, we are aiming to compare the effectiveness and safety of the non-vitamin K antagonist oral anticoagulants (NOACs) and warfarin in AF patients with HF and preserved (HFpEF), mildly reduced (HFmrEF), and reduced (HFrEF) ejection fraction.

**Methods and results:**

We systematically searched the PubMed, Cochrane, and Embase databases until January 2022. The primary effectiveness and safety outcomes were stroke or systemic embolism (SSE) and major bleeding, respectively. We abstracted risk ratios (RR) and 95% confidence intervals (CIs) and compiled them using a random-effects model. We analyzed data of 266,291 patients from 10 studies. By comparing NOACs with warfarin, patients with AF and HF have reduced the risk of SSE (RR: 0.83, 95% CI 0.76–0.91), all-cause mortality (RR: 0.85, 95% CI 0.80–0.91), major bleeding (RR: 0.79, 95% CI 0.69–0.90), and intracranial hemorrhage (RR: 0.54, 95% CI 0.46–0.63). Further analyses based on the HF subtypes showed that NOACs reduced the chances of SSE (RR: 0.71, 95% CI 0.53–0.94) in the HFrEF group and major bleeding (RR: 0.74, 95% CI 0.57–0.95) in HFmrEF and HFpEF groups. There were no differences regarding SSE (RR: 0.91, 95% CI 0.76–1.09) in HFmrEF and HFpEF groups and major bleeding (RR: 0.99, 95% CI 0.79–1.23) in the HFrEF group.

**Conclusion:**

For patients with AF and HF, NOACs have better or similar effectiveness and safety than warfarin, but the stroke prevention superiority of NOACs over warfarin varies in different HF subtypes.

## Introduction

As the most frequent sustained cardio rhythm disorder, atrial fibrillation (AF) frequently exists alongside heart failure (HF) and is linked to a higher risk of stroke and all-cause mortality (1). Anticoagulant therapy, an essential component of the integrated Atrial fibrillation Better Care (ABC) pathway in patients with AF, has been demonstrated to reduce the potential adverse outcomes (2). Current guidelines consistently recommend non-vitamin K antagonist oral anticoagulants (NOACs) as a priority of anticoagulants for patients with AF (3, 4). Traditionally, HF was divided into two phenotypes: HF with reduced (HFrEF) or preserved EF (HFpEF) ejection fraction (EF) (5). Recently, the European Society of Cardiology (ESC) recommends three HF subtypes: HF and preserved (HFpEF, EF ≥ 50%), mildly reduced (HFmrEF, EF 41–49%), and reduced (HFrEF, EF ≤ 40%) EF (6, 7). Although HFrEF and HFpEF share some similar clinical manifestations, they represent entirely different diseases in the HF spectrum, and they are studied and treated separately (8).

For patients in conjunction with AF and HF, some randomized controlled trial (RCT) *post hoc* analyses have shown that NOACs are non-inferior or even better than vitamin-K antagonists (VKAs) in terms of effectiveness and safety (9–12). An earlier meta-analysis by Chen et al. demonstrated that compared to warfarin, NOACs led to significantly fewer stroke or systemic embolism (SSE) and major bleeding risks in patients with concomitant AF and HF (13). The American Heart Association’s scientific statement encouraged a decision-making process for AF and HFrEF including guideline-directed HF treatment therapy, lifestyle, risk factor adjustment, oral anticoagulation based on the CHAD2DS2-VASc score, pharmacological rate control, and cardioversion if necessary (including catheter ablation and antiarrhythmic treatment) (14). As for AF and HFpEF, there is still a lack of corresponding guidelines and clinical evidence. In addition, a comparison of NOACs and VKAs in AF patients with different HF subtypes (HFpEF, HFmrEF, and HFrEF) remain unknown. Therefore, our study evaluated the safety and effectiveness of NOACs against VKAs in patients with AF accompanied by HF, especially in different subtypes of HF.

## Methods

We conducted the meta-analysis based on the Cochrane Systematic Review Handbook (15), and the writing followed the statement of Preferred Reporting Items for Systematic Reviews and Meta-Analyses (PRISMA) (16) (list of checkpoints displayed in Supplementary Table 1). The included studies were reviewed by the relevant ethics committee before publication, so we did not need ethical approval.

### Criteria for study eligibility

For our analysis, the following criteria were used to select studies: (1) population: adult patients with non-valvular AF complicated with HFpEF, HFmrEF, or HFrEF; (2) outcome measures and intervention: studies assessing at least one effectiveness or safety outcome of NOACs (edoxaban, rivaroxaban, apixaban, or dabigatran) versus VKAs; and (3) study design: RCTs and observational (prospective or retrospective cohort) studies.

We excluded studies where cross-sections, reviews, reports on cases, editorials, letters, or meeting abstracts had insufficient data or study details. For studies that met our inclusion criteria but had overlapping populations, our priority was to study long-term follow-ups or large sample sizes.

### Search strategy

We systematically searched all the studies published on electronic databases, such as Cochrane Library, Embase, and PubMed, without linguistic limits (up to January 2022). Supplementary Table 2 presents a listing of the retrieval strategies used: (1) heart failure, AND (2) atrial fibrillation OR atrial flutter, AND (3) non-vitamin K antagonists OR direct oral anticoagulants OR novel oral anticoagulants OR new oral anticoagulants OR edoxaban OR apixaban OR rivaroxaban OR dabigatran, and (4) acenocoumarol OR warfarin OR coumadin OR phenprocoumon OR indandione OR vitamin-K antagonists OR phenindione OR anisindione.

### Selection of studies and data extraction

A team of two reviewers reviewed all retrieved studies and abstracted relevant data independently. Based on the qualifications for inclusion, we reviewed the titles and abstracts of the studies and then read the full text in detail to determine the truly eligible studies. In the case of conflict between two reviewers, we reached a consensus by consulting with a third reviewer. We collected the following data from the studies we included: author, publication year, country of the population, data source, study duration, study design, demographics of patients, follow-up period, types of NOACs and dosages, and outcome data (size of sample, count of events in a group, and adjusted effect estimates).

### Study outcomes

The effectiveness outcomes included SSE, all-cause death, and ischemic stroke, whereas major bleeding, gastrointestinal bleeding, and intracranial bleeding were the safety outcomes. SSE and major bleeding were the primary effectiveness and safety outcomes, whereas others were the secondary outcomes. All the outcomes included in this meta-analysis and definitions of the primary outcomes are shown in Supplementary Table 3.

### Quality assessment

The Newcastle-Ottawa Scale (NOS) items were used to evaluate observational studies. The RCT *post hoc* analyses were used as an observational study for quality evaluation. A total of nine points were allocated to the NOS tool’s three domains: cohort selection (0–4 points), cohort comparability (0–2 points), and outcome assessment (0–3 points). NOS scores of 6 or more points were considered medium to high quality, and a score below six points was regarded as low quality (17).

### Statistical analysis

Cochrane *Q* test and *I*^2^ values were used to determine heterogeneity between studies in statistical terms. A *p*-value of < 0.1 or *I*^2^ value > 50% indicated significant heterogeneity across studies. The study effect was estimated with adjusted risk ratios (RRs) and 95% confidence intervals (CIs). The RR natural logarithm and its corresponding standard deviation ((Ln[upper CI]-Ln[lower CI])/3.92) were calculated. Because there were different types and doses of NOACs included in this study, the random-effects model was used in conjunction with the inverse variance method to pool the natural logarithms. Subgroups were performed based on taking 40 and 50% as the left ventricular ejection fraction (LVEF) boundary, study type, renal function, CHA2DS2-VASc score, types of NOACs, New York Heart Association (NYHA) class, and follow-up time. The bias of publication was examined by visually inspecting the funnel plots in which the logRRs were plotted against their standard errors. In addition, Egger’s and Begg’s tests for each outcome were applied to examine publication bias.

Review Manager version 5.4 (the Cochrane Collaboration 2014, Rigshospitalet, Nordic Cochrane Centre Copenhagen, Denmark) was used to perform all the statistical analyses. *p-*values of < 0.05 were considered statistically significant.

## Results

### Study identification and selection

**FIGURE 1:**
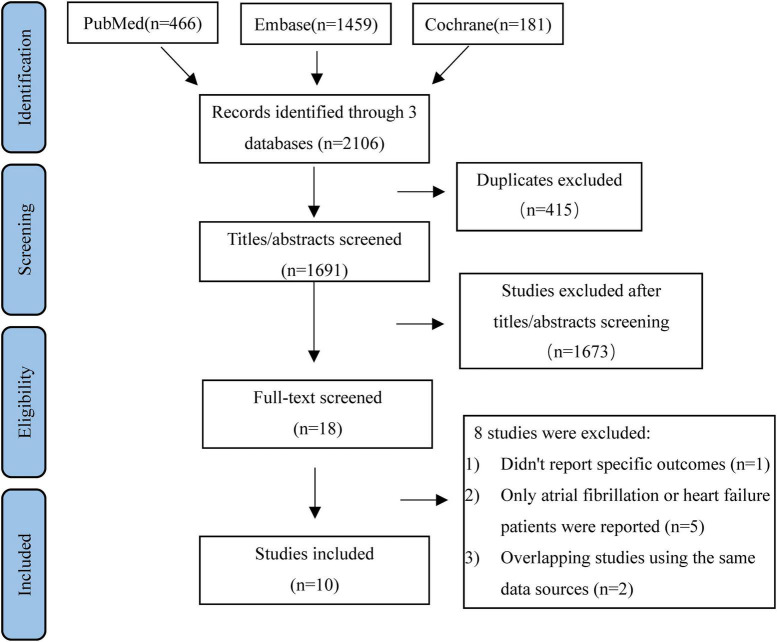
Flowchart of studies identified, screened, excluded, and included in the meta-analysis.

The literature retrieval flowchart is presented in Figure 1. We identified 2,106 articles using the PubMed, Embase, and Cochrane Library databases through our search strategy. A total of 415 studies were duplicated, and 1,691 articles were excluded after screening the title and abstract. The remaining 18 studies were assessed by reading the full text and eight articles were removed for eligibility. Finally, our meta-analysis included 10 studies (4 RCT *post hoc* analyses and six observational studies) comprising 266,291 patients (9–12, 18–23).

### Study characteristics

A summary of study characteristics at baseline is shown in Table 1. Among them, four studies were *post hoc* analyses of RCTs, including RE-LY (dabigatran), ROCKET AF (rivaroxaban), ARISTOTLE (apixaban), and ENGAGE AF-TIMI 48 (edoxaban) trials (9–12). The other 6 studies were observational studies from the United States (*n* = 4) (18, 19, 21, 22), Japan (*n* = 1) (23), and Sweden (*n* = 1) (20), respectively. Sample sizes ranged from 4,904 to 49,448 patients, and the duration of median follow-up time was 0.4–2.8 years. The definition of HF was extracted from the originally included studies and shown in Supplementary Table 3. As a measure of quality, the NOS tool was used to assess the included studies, all of which were judged to be medium-to-high and deemed qualified (Supplementary Table 4).

**TABLE 1:** Patients’ characteristics of the selected studies for this meta-analysis.

References	Location	Database source	Age (years)	CHA2DS2-VASc score	HAS-BLED score	OAC	Antiplatelet agents, n (%)	NYHA class III or IV, n (%)	Follow-up Period (y)	LVEF subgroup boundary	Outcomes used in this meta-analysis
(12)	Multinational	ENGAGE AF-TIMI 48 trial, 11/2008–11/2010; *post hoc* analysis of RCT	75	4.5	2.4	EDO warfarin	2,437 (29.9) NA	1,801 (13)	2.8	<50% (*n* = 3,103) ≥50% (*n* = 3,236)	SSE, MB
(11)	Multinational	ARISTOTLE trial, 12/2006–04/2010; *post hoc* analysis of RCT	71	NA	NA	API warfarin	2,089 (35.2) NA	1,335 (22.5)	1.5	≤40% (*n* = 3,207) >40% (*n* = 2,736)	SSE, IS, All-cause death, MB, HS, GIB
(10)	Multinational	ROCKET AF trial, 12/2006–06/2009; *post hoc* analysis of RCT	74	5.1	NA	RIV warfarin	1,373 (30.3) 1,428 (31.7)	1,329 (30.0) 1,316 (29.9)	1.9	<40% (*n* = 2,145) ≥40% (*n* = 6,888)	SSE, MB
(9)	Multinational	RE-LY trial, 12/2005–12/2007; *post hoc* analysis of RCT	73	NA	NA	DA warfarin	NA NA	NA	2.0	≤40% (*n* = 1,258) >40% (*n* = 1,631)	SSE, MB
(18)	United States	HealthCore Integrated Research Environment, 11/2009–01/2016; retrospective cohort	70	3.3	2.1	DA RIV API warfarin	1,699 (19.9) 745 (20.2) 1,722 (20.5) 4,733 (20.2)	NA	0.4*/0.5	NA	MB
(20)	Sweden	Cross-linked national registers, 12/2011–12/2014; retrospective cohort	74	3.3	NA	NOACs warfarin	2,367 (12.7) 7,215 (14.6)	NA	0.7/1.7*	NA	SSE, All-cause death, MB
(22)	United States	Truven MarketScan Commercial and Medicare supplemental database, 11/2011–12/2016; retrospective cohort	74	4.0	2.0	RIV warfarin	578 (16.9) 612 (17.9)	NA	1.4	NA	SSE, IS, MB, ICH
(23)	Japan	Fukushima Medical University Hospital, 2011-015; retrospective cohort	70	4.3–4.4	2.7–2.8	NOACs warfarin	108 (42.0) 62 (41.3)	8 (3.1) 4 (2.7)	3.0	<50% (*n* = 127) ≥50% (*n* = 101)	All-cause death
(19)	United States	The Center for Medicare and Medicaid Services, 01/2012–09/2016; retrospective cohort	79–80	5.2–5.4	3.5–3.7	DA RIV API warfarin	887 (20.64) 3,788 (24.11) 2,786 (26.36) NA	NA	0.6	NA	SSE, IS, All-cause death, MB, ICH, GIB
(21)	United States	Veterans Administration databases, 10/2010–08/2017; retrospective cohort	72	4.1	3.37–3.57	NOACs warfarin	10,561 (40.9) 9,548 (40.4)	NA	1.4*/1.5	<40% ≥40%	All-cause death, MB, GIB

Data were presented as mean for age, CHA2DS2-VASc score, HAS-BLED score and follow-up period; *: represents the median follow-up time of warfarin group, when there are two follow-up times. CHA2DS2-VASc, Congestive heart failure/left ventricular ejection fraction ≤ 40%, Hypertension, age 75 years of age and older, Diabetes mellitus, Stroke/transient ischemic attack/thromboembolism history, Vascular disease, Age 65–74 years, Sex (female); HAS-BLED, Hypertension, Abnormal liver/renal function, Stroke, Bleeding history or predisposition, Labile international normalized ratio, Elderly, Drugs/alcohol concomitantly; OAC, oral anticoagulants; LVEF, left ventricular ejection fraction; NOACs, non-vitamin K antagonist oral anticoagulants; DA, dabigatran; RIV, rivaroxaban; API, apixaban; SSE, stroke or systemic embolism; MB, major bleeding; IS, ischemic stroke; ICH, intracranial hemorrhage; GIB, gastrointestinal bleeding; NOS, Newcastle-Ottawa Scale; NA, not available.

### Effectiveness and safety outcomes in atrial fibrillation with and without heart failure population

Among AF with HF patients, in comparison with warfarin (Supplementary Figure 1), the use of NOACs was linked to lower risks of SSE (RR: 0.83, 95% CI 0.76–0.91) and all-cause death (RR: 0.85, 95% CI 0.80–0.91), while a significant difference was not observed in ischemic stroke (RR: 0.88, 95% CI 0.74–1.04). As for the safety outcomes compared to warfarin (Supplementary Figure 2), NOACs in patients with AF and HF were found to reduce major bleeding (RR: 0.79, 95% CI 0.60–0.90) and intracranial bleeding (RR: 0.54, 95% CI 0.46–0.63) risks significantly, but the risk of gastrointestinal bleeding (RR: 1.00, 95% CI 0.76–1.31) was not different between the two groups.

The effectiveness and safety of NOACs and warfarin in AF patients without HF were consistent with those in patients with AF and HF. In AF patients without HF (Supplementary Figure 3), NOACs reduced the risk of SSE (RR: 0.83, 95% CI 0.71–0.97), all-cause mortality (RR: 0.85, 95% CI 0.78–0.92), major bleeding (RR: 0.77, 95% CI 0.68–0.89) and intracranial hemorrhage (RR: 0.46, 95% CI 0.35–0.61) than warfarin, but there was no significant difference in the risks of ischemic stroke (RR: 0.91, 95% CI 0.74–1.12) and gastrointestinal bleeding (RR: 1.08, 95% CI 0.72–1.64).

### Effectiveness and safety outcomes in different heart failure subtypes

**FIGURE 2:**
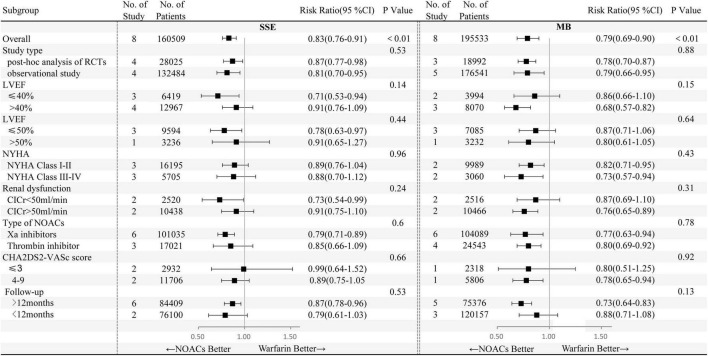
Primary effectiveness and safety outcomes of non-vitamin K antagonist oral anticoagulants (NOACs) versus warfarin according to different subgroups. NYHA, New York Heart Association; ClCr, creatinine clearance; CI, confidence interval.

Effects of NOACs on primary effectiveness and safety outcomes in HFrEF and HFpEF subgroups were analyzed taking 40 and 50% as the LVEF boundary, respectively (Figure 2). Compared with warfarin, the use of NOACs was related to lower SSE risks in patients with HFrEF independent of the LVEF boundary of 40 or 50%.

**FIGURE 3:**
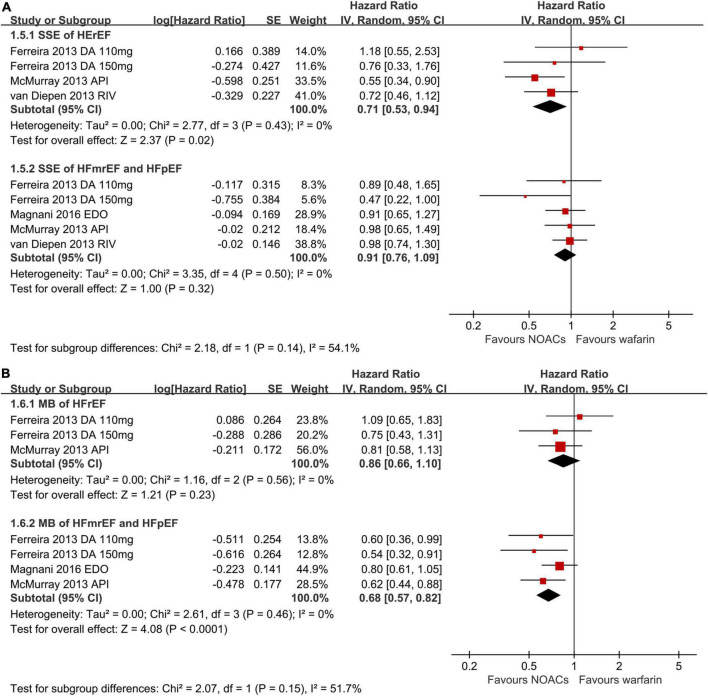
Forest plot for primary effectiveness **(A)** and safety **(B)** outcomes in HFrEF, HFmrEF, and HFpEF. HFrEF, heart failure with reduced ejection fraction; HFmrEF, heart failure with mildly reduced ejection fraction; HFpEF, heart failure with preserved ejection fraction; CI, confidence interval.

When categorizing HF into different types of HFpEF, HFmrEF, and HFrEF, NOACs against warfarin significantly decreased SSE (RR: 0.71, 95% CI 0.53–0.94) risks in patients with AF and HFrEF (Figure 3A). However, no significant statistical difference in the risks of SSE (RR: 0.91, 95% CI 0.76–1.09) was indicated in AF patients with HFmrEF or HFpEF. As presented in Figure 3B, in AF patients with concomitant HFmrEF or HFpEF, as compared to warfarin, NOACs reduced the risk of major bleeding (RR: 0.68, 95% CI 0.57–0.82), whereas major bleeding (RR: 0.86, 95% CI 0.66–1.10) risks did not differ in patients with AF and HFrEF.

### Subgroup analyses

Subgroup analyses were performed based on study type (RCT *post hoc* analysis and observational study), class of NYHA (NYHA I-II and NYHA III-IV), renal function (creatinine clearance was 50 ml/min as the boundary), CHA2DS2-VASc score (≤3, 4–9), types of NOACs (factor Xa inhibitors, thrombin inhibitor), and follow-up time (12 months as the boundary) (Figure 2).

In comparison with warfarin users, lower SSE and major bleeding risks were associated with factor Xa inhibitor, whereas thrombin inhibitor users had smaller major bleeding risks and similar SSE risks. In addition, during long-term follow-up (>12 months), NOACs versus warfarin significantly decreased the risks of SSE and major bleeding. In other subgroup analyses based on NYHA class, renal dysfunction, study type, LVEF with 50% as the boundary, and the CHA2DS2-VASc score, NOACs and warfarin were at least as safe and effective as each other for the prevention of strokes.

### Bias in publication

Publication bias was evaluated through a visual check of the asymmetry of the funnel plots (Supplementary Figures 4, 5). No obvious publication biases were found for SSE, ischemic stroke, all-cause mortality, and major bleeding. Egger’s and Begg’s tests did not indicate publication biases for the primary outcomes. However, the funnel plot for intracranial hemorrhage or gastrointestinal bleeding was asymmetrical possibly because only a few studies were included in terms of these outcomes. Therefore, the pooled data should be interpreted cautiously.

## Discussion

We evaluated the adverse outcomes of NOACs across different HF subtypes by performing a meta-analysis in this study. We found that in comparison with warfarin, NOACs use was significantly linked to reduced risks of SSE, all-cause mortality, intracranial bleeding, and major bleeding, whereas risks of ischemic stroke and gastrointestinal bleeding did not differ significantly between the treatment groups. In addition, NOACs outweighed warfarin in decreasing the risks of SSE in the HFrEF group and major bleeding in HFmrEF or HFpEF groups.

The coexistence of AF and HF was common with a patient prevalence of AF in HF exceeding 20% (24). It has been reported that SSE and all-cause mortality risks were increased when both conditions were present (11). As recommended by the current guidelines, NOACs are more effective and safer than warfarin in stroke prevention for AF patients (25). In this meta-analysis, we found that for patients with AF and HF, NOACs were also superior to warfarin in the reduction of SSE, all-cause mortality, intracranial bleeding, and major bleeding. This was consistent with prior meta-analyses which demonstrated that despite the increasing death rate among patients with HF and AF, SSE, major, and intracranial bleeding in AF patients with concomitant HF were significantly reduced by NOACs compared with warfarin (13, 26).

The prevalence of AF and prognosis vary across different HF subtypes. According to the ESC heart failure long-term registry, the prevalence of AF increases with the increase of LVEF (HFrEF: 27%, HFmrEF: 29%, and HFpEF: 39%) (27). Patients with HFpEF are usually older, more likely to be women, and usually have multiple comorbidities, including hypertension, obesity, and diabetes, making the CHA2DS2-VASc score much higher than those with HFrEF (28). However, the annual incidence of stroke was linearly increasing by 0.054% per each 1% of LVEF decrease (29). Indeed, patients with HFrEF had the highest risks of stroke and mortality despite a relatively lower CHA2DS2-VASc score compared with HFpEF (29). In our meta-analysis, NOACs were linked to reduced SSE (RR: 0.71, 95% CI 0.53–0.94) risks significantly in AF patients with HFrEF but not those with HFmrEF or HFpEF. However, limited evidence was available in terms of the superiority of NOACs over warfarin in patients with AF and different phenotypes of HF. Further robust clinical trials were warranted to investigate the safety and efficacy of NOACs in patients with AF among different phenotypes (11, 12).

In addition, the definition of HF and the cut-off value of HFpEF, HFmrEF, and HFrEF were also heterogeneous. Therefore, the results derived from the included studies may not reflect the real therapeutic effects of NOACs and should be interpreted cautiously.

### Limitations

Our meta-analysis had several limitations that should be further addressed. First, the choice of drugs for these patients depends on many factors, and it is difficult to directly compare NOACs with each other given the differences in trial design and study population among the four *post hoc* analyses of RCTs. Second, the definition of HF and the cut-off valve of HFpEF, HFmrEF, and HFrEF differ in the included studies in this meta-analysis, hence the results should be interpreted cautiously. Further robust clinical trials with consistent definitions and categories of HF are warranted.

## Conclusion

Our current evidence of this meta-analysis suggested that in patients with AF and HF, NOACs have better or similar effectiveness and safety than warfarin, but the stroke prevention superiority of NOACs over warfarin varies in different HF subtypes.

## Data availability statement

The original contributions presented in this study are included in the article/Supplementary material, further inquiries can be directed to the corresponding authors.

## Author contributions

JH and ZW designed the study and revised the manuscript. KW and ZX carried out the literature search, article screen, assessing quality, and statistics. KW wrote the manuscript. ZW and YC reformulated the manuscript and revised the English grammar. All authors contributed to the article and approved the submitted version.
