# Supplementary material for: Non-vitamin K antagonist oral anticoagulants versus warfarin in atrial fibrillation patients with heart failure and preserved, mildly reduced, and reduced ejection fraction: A systemic review and meta-analysis
Source: Front Cardiovasc Med. 2022 Jul 29;9:949726. doi: 10.3389/fcvm.2022.949726 (PMC9372303; doi:10.3389/fcvm.2022.949726)
Supplement: Supplementary file 1 [file Data_Sheet_1.PDF]

SUPPLEMENTAL MATERIAL

Supplementary Table 1. PRISMA (Preferred Reporting Items for Systematic Reviews and Meta-Analyses) 2020 Checklist

Section and Topic	Item #	Checklist item	Location where item is reported
**TITLE**			
Title	1	Identify the report as a systematic review.	Page 1
**ABSTRACT**			
Abstract	2	See the PRISMA 2020 for Abstracts checklist.	Page 3
**INTRODUCTION**			
Rationale	3	Describe the rationale for the review in the context of existing knowledge.	Page 5
Objectives	4	Provide an explicit statement of the objective(s) or question(s) the review addresses.	Page 5
**METHODS**			
Eligibility criteria	5	Specify the inclusion and exclusion criteria for the review and how studies were grouped for the syntheses.	Page 6
Information sources	6	Specify all databases, registers, websites, organisations, reference lists and other sources searched or consulted to identify studies. Specify the date when each source was last searched or consulted.	Page 8
Search strategy	7	Present the full search strategies for all databases, registers and websites, including any filters and limits used.	Page 6
Selection process	8	Specify the methods used to decide whether a study met the inclusion criteria of the review, including how many reviewers screened each record and each report retrieved, whether they worked independently, and if applicable, details of automation tools used in the process.	Page 7
Data collection process	9	Specify the methods used to collect data from reports, including how many reviewers collected data from each report, whether they worked independently, any processes for obtaining or confirming data from study investigators, and if applicable, details of automation tools used in the process.	Page 7
Data items	10a	List and define all outcomes for which data were sought. Specify whether all results that were compatible with each outcome domain in each study were sought (e.g. for all measures, time points, analyses), and if not, the methods used to decide which results to collect.	Page 6
	10b	List and define all other variables for which data were sought (e.g. participant and intervention characteristics, funding sources). Describe any assumptions made about any missing or unclear information.	Page 7
Study risk of bias assessment	11	Specify the methods used to assess risk of bias in the included studies, including details of the tool(s) used, how many reviewers assessed each study and whether they worked independently, and if applicable, details of automation tools used in the process.	Page 7
Effect measures	12	Specify for each outcome the effect measure(s) (e.g. risk ratio, mean difference) used in the synthesis or presentation of results.	Page 6

Section and Topic	Item #	Checklist item	Location where item is reported
Synthesis methods	13a	Describe the processes used to decide which studies were eligible for each synthesis (e.g. tabulating the study intervention characteristics and comparing against the planned groups for each synthesis (item #5)).	Table 1
	13b	Describe any methods required to prepare the data for presentation or synthesis, such as handling of missing summary statistics, or data conversions.	Page 7
	13c	Describe any methods used to tabulate or visually display results of individual studies and syntheses.	Page 7
	13d	Describe any methods used to synthesize results and provide a rationale for the choice(s). If meta-analysis was performed, describe the model(s), method(s) to identify the presence and extent of statistical heterogeneity, and software package(s) used.	Page 7
	13e	Describe any methods used to explore possible causes of heterogeneity among study results (e.g. subgroup analysis, meta-regression).	Page 7
	13f	Describe any sensitivity analyses conducted to assess robustness of the synthesized results.	Page 7
Reporting bias assessment	14	Describe any methods used to assess risk of bias due to missing results in a synthesis (arising from reporting biases).	None
Certainty assessment	15	Describe any methods used to assess certainty (or confidence) in the body of evidence for an outcome.	Page 7
**RESULTS**			
Study selection	16a	Describe the results of the search and selection process, from the number of records identified in the search to the number of studies included in the review, ideally using a flow diagram.	Figure 1
	16b	Cite studies that might appear to meet the inclusion criteria, but which were excluded, and explain why they were excluded.	Page 8
Study characteristics	17	Cite each included study and present its characteristics.	Page 8
Risk of bias in studies	18	Present assessments of risk of bias for each included study.	Page 6
Results of individual studies	19	For all outcomes, present, for each study: (a) summary statistics for each group (where appropriate) and (b) an effect estimate and its precision (e.g. confidence/credible interval), ideally using structured tables or plots.	Page 8
Results of syntheses	20a	For each synthesis, briefly summarise the characteristics and risk of bias among contributing studies.	Page 7
	20b	Present results of all statistical syntheses conducted. If meta-analysis was done, present for each the summary estimate and its precision (e.g. confidence/credible interval) and measures of statistical heterogeneity. If comparing groups, describe the direction of the effect.	Page 8
	20c	Present results of all investigations of possible causes of heterogeneity among study results.	Page 9
	20d	Present results of all sensitivity analyses conducted to assess the robustness of the synthesized results.	Page 9

Section and Topic	Item #	Checklist item	Location where item is reported
Reporting biases	21	Present assessments of risk of bias due to missing results (arising from reporting biases) for each synthesis assessed.	None
Certainty of evidence	22	Present assessments of certainty (or confidence) in the body of evidence for each outcome assessed.	None
**DISCUSSION**			
Discussion	23a	Provide a general interpretation of the results in the context of other evidence.	Page 10
	23b	Discuss any limitations of the evidence included in the review.	Page 11
	23c	Discuss any limitations of the review processes used.	Page 11
	23d	Discuss implications of the results for practice, policy, and future research.	Page 11
**OTHER INFORMATION**			
Registration and protocol	24a	Provide registration information for the review, including register name and registration number, or state that the review was not registered.	None
	24b	Indicate where the review protocol can be accessed, or state that a protocol was not prepared.	None
	24c	Describe and explain any amendments to information provided at registration or in the protocol.	None
Support	25	Describe sources of financial or non-financial support for the review, and the role of the funders or sponsors in the review.	Page 12
Competing interests	26	Declare any competing interests of review authors.	Page 12
Availability of data, code and other materials	27	Report which of the following are publicly available and where they can be found: template data collection forms; data extracted from included studies; data used for all analyses; analytic code; any other materials used in the review.	Contact the corresponding author

From: Page MJ, McKenzie JE, Bossuyt PM, Boutron I, Hoffmann TC, Mulrow CD, et al. The PRISMA 2020 statement: an updated guideline for reporting systematic reviews. BMJ 2021;372:n71. doi: 10.1136/bmj.n71.For more information, visit: http://www.prisma-statement.org/

Supplementary Table 2. Search strategies in the PubMed, Embase, and the Cochrane Library

	Queries	Items
**PubMed**		
#1	heart failure AND (atrial fibrillation OR atrial flutter)	16215
#2	non-vitamin K antagonists OR direct oral anticoagulants OR new oral anticoagulants OR novel oral anticoagulants OR dabigatran OR rivaroxaban OR apixaban OR edoxaban	21609
#3	vitamin-K antagonists OR coumadin OR warfarin OR phenprocoumon OR acenocoumarol OR indandione OR fluindione OR phenindione OR anisindione	42778
#4	#1 and #2 and #3 to present	466
**Embase**		
#1	'heart failure' AND ('atrial fibrillation' OR 'atrial flutter')	45092
#2	'non-vitamin K antagonists' OR 'direct oral anticoagulants' OR 'new oral anticoagulants' OR 'novel oral anticoagulants' OR dabigatran OR rivaroxaban OR apixaban OR edoxaban	40304
#3	'vitamin-K antagonists' OR coumadin OR warfarin OR phenprocoumon OR acenocoumarol OR indandione OR fluindione OR phenindione OR anisindione	119186
#4	#1 and #2 and #3 to present	1459
**Cochrane**		
#1	heart failure AND (atrial fibrillation OR atrial flutter)	3214
#2	non-vitamin K antagonists OR direct oral anticoagulants OR new oral anticoagulants OR novel oral anticoagulants OR dabigatran OR rivaroxaban OR apixaban OR edoxaban	4567
#3	vitamin-K antagonists OR coumadin OR warfarin OR phenprocoumon OR acenocoumarol OR indandione OR fluindione OR phenindione OR anisindione	6259
#4	#1 and #2 and #3 to present	181

Supplementary Table 3. Definitions of the primary efficacy and safety outcomes and hreat failure in this meta-analysis

Study	Outcomes included for analysis	Definitions of the primary outcomes		Definition of heart failure
		Stroke or systemic embolism	Major bleeding	
Magnani-2016	SSE, IS, All-cause death, MB, HS, GIB	Stroke (ischaemic or hemorrhagic) or systemic embolic events	MB(ISTH)	NYHA class and LVEF: the presence or previous history of HF stage C or D according to the ACC/AHA definition
McMurray-2013	SSE, IS, All-cause death, MB, HS, GIB	Stroke (ischemic or hemorrhagic) or systemic embolism	MB(ISTH)	Symptomatic HF, LVSD, and LVEF: (1) patients with LVSD, with or without symptomatic HF;(2) patients with HF and preserved EF (>40%), normal LV function, or mild LVSD
van Diepen-2013	SSE, MB	Stroke (ischemic or hemorrhagic) or noncentral nervous system embolism	Major or non-major clinically relevant bleeding	Symptomatic HF and LVEF: a history of HF or a left ventricular EF <40%
Ferreira-2013	SSE, MB	Stroke (including hemorrhagic) or systemic embolism	MB(ISTH)	Symptomatic HF and NYHA class: the presence of NYHA class II or higher HF symptoms (fatigue, dyspnoea)
Adeboyeje G-2017	MB	NA	Intracranial, GI, and other bleeding	ICD-9 codes: 402.01, 402.11, 402.91, 404.01, 404.03, 404.11, 404.13, 404.91, 404.93, 428.xx
Friberg L-2017	SSE, All cause death, MB	Stroke or systemic embolism	Intracranial bleeding, GIB, Other bleeding	ICD-10 codes: I50, I110, I130, I132, I255, K761, I42-43
Martinez B K-2019	SSE, IS, MB, ICH	Stroke or systemic embolism	MB(Cunningham algorithm)	ICD-10 codes: I50, I09.81
Yoshihisa A-2018	All cause death	NA	NA	Symptomatic HF: hospitalized with decompensated HFdefined based on the Framingham criteria
Amin A-2019	SSE, IS, All cause death, MB, ICH, GIB	Stroke or systemic embolism	GIB, IB, other MB	ICD-9 codes: 428.0, 428.1, 428.2, 428.3, 428.4, 428.9
Jackevicius C A-2021	All cause death, MB, GIB	SSE	MB(Cunningham algorithm)	ICD-9 428.xx/ICD-10 I50.x, I11.0, I13.0.2

SSE, Stroke or systemic embolism; IS, Ischemic stroke; MB, Major bleeding; HS, Haemorrhagic stroke;ICH, intracranial hemorrhage; GIB, Gastrointestinal bleeding; ISTH, International Society on Thrombosis and Haemostasis; HF, heart failure; NYHA, New York Heart Associ-ation; LVEF, eft ventricular ejection fraction; ACC, American College of Cardiology; AHA, American Heart Association; LVSD, left ventricular systemic dysfunction;ICD, International Classification of Diseases; NA, not available.

Supplementary Table 4. Quality assessment of included studies based on the NOS tool

Studies	Selection				Comparability	Outcome			Total
	**Exposed cohort**	**Non-exposed cohort**	**Ascertainment of exposure**	**Outcome of interest**		**Assessment of outcome**	**Length of follow-up**	**Adequacy of follow up**	
Magnani-2016	*	*	*		**	*	*	*	**8**
McMurray-2013	*	*	*		**	*	*	*	**8**
van Diepen-2013	*	*	*		**	*	*	*	**8**
Ferreira-2013	*	*	*		**	*	*	*	**8**
Adeboyeje G-2017	*	*	*		**	*		*	**7**
Friberg L-2017	*	*	*		**	*		*	**7**
Martinez B K-2019	*	*	*		**	*	*	*	**8**
Yoshihisa A-2018	*	*	*		**	*	*	*	**8**
Amin A-2019	*	*	*		**	*		*	**7**
Jackevicius C A-2021	*	*	*		**	*	*	*	**8**

Study or Subgroup	log[Risk Ratio]	SE	Weight	Risk Ratio IV, Random, 95% CI
**1.1.1 SSE**				
Amin 2019 API	-0.446	0.146	8.3%	0.64 [0.48, 0.85]
Amin 2019 DA	-0.073	0.204	4.8%	0.93 [0.62, 1.39]
Amin 2019 RIV	-0.431	0.113	12.1%	0.65 [0.52, 0.81]
Ferreira 2013 DA 110mg	-0.01	0.184	5.7%	0.99 [0.69, 1.42]
Ferreira 2013 DA 150mg	-0.288	0.196	5.2%	0.75 [0.51, 1.10]
Friberg 2017 mix	0.058	0.136	9.3%	1.06 [0.81, 1.38]
Jackevicius 2021 API	-0.186	0.149	8.1%	0.83 [0.62, 1.11]
Jackevicius 2021 DA	-0.117	0.219	4.2%	0.89 [0.58, 1.37]
Jackevicius 2021 RIV	-0.051	0.234	3.8%	0.95 [0.60, 1.50]
Mangani 2016 EDO[NYHA I-II]	-0.128	0.124	10.6%	0.88 [0.69, 1.12]
Mangani 2016 EDO[NYHA III-IV]	-0.186	0.209	4.6%	0.83 [0.55, 1.25]
Martinez 2019 RIV	-0.198	0.286	2.6%	0.82 [0.47, 1.44]
McMurray 2013 API[HFpEF]	-0.02	0.212	4.5%	0.98 [0.65, 1.49]
McMurray 2013 API[LVSD]	-0.598	0.251	3.3%	0.55 [0.34, 0.90]
van Diepen 2013 RIV	-0.094	0.108	12.8%	0.91 [0.74, 1.12]
**Subtotal (95% CI)**			**100.0%**	**0.83 [0.76, 0.91]**
Heterogeneity: Tau² = 0.01; Chi² = 17.27, df = 14 (P = 0.24); I² = 19%				
Test for overall effect: Z = 3.85 (P = 0.0001)				
**1.1.2 IS**				
Amin 2019 API	-0.223	0.17	15.9%	0.80 [0.57, 1.12]
Amin 2019 DA	0.215	0.236	10.0%	1.24 [0.78, 1.97]
Amin 2019 RIV	-0.386	0.131	21.5%	0.68 [0.53, 0.88]
Mangani 2016 EDO[NYHA I-II]	0	0.139	20.2%	1.00 [0.76, 1.31]
Mangani 2016 EDO[NYHA III-IV]	-0.01	0.23	10.4%	0.99 [0.63, 1.55]
Martinez 2019 RIV	-0.261	0.324	5.9%	0.77 [0.41, 1.45]
McMurray 2013 API[HFpEF]	0.157	0.249	9.2%	1.17 [0.72, 1.91]
McMurray 2013 API[LVSD]	-0.431	0.296	6.9%	0.65 [0.36, 1.16]
**Subtotal (95% CI)**			**100.0%**	**0.88 [0.74, 1.04]**
Heterogeneity: Tau² = 0.02; Chi² = 9.89, df = 7 (P = 0.20); I² = 29%				
Test for overall effect: Z = 1.53 (P = 0.13)				
**1.1.3 All cause death**				
Amin 2019 API	-0.315	0.045	10.1%	0.73 [0.67, 0.80]
Amin 2019 DA	-0.151	0.068	8.1%	0.86 [0.75, 0.98]
Amin 2019 RIV	-0.174	0.033	11.1%	0.84 [0.79, 0.90]
Friberg 2017 mix	-0.08	0.06	8.8%	0.92 [0.82, 1.04]
Jackevicius 2021 API	-0.274	0.03	11.3%	0.76 [0.72, 0.81]
Jackevicius 2021 DA	-0.315	0.045	10.1%	0.73 [0.67, 0.80]
Jackevicius 2021 RIV	-0.083	0.056	9.2%	0.92 [0.82, 1.03]
Mangani 2016 EDO[NYHA I-II]	-0.051	0.073	7.7%	0.95 [0.82, 1.10]
Mangani 2016 EDO[NYHA III-IV]	0.02	0.117	4.8%	1.02 [0.81, 1.28]
McMurray 2013 API[HFpEF]	-0.117	0.126	4.4%	0.89 [0.69, 1.14]
McMurray 2013 API[LVSD]	-0.02	0.109	5.3%	0.98 [0.79, 1.21]
van Diepen 2013 RIV	-0.073	0.068	8.1%	0.93 [0.81, 1.06]
Yoshihisa 2018 mix	-0.642	0.314	1.0%	0.53 [0.28, 0.97]
**Subtotal (95% CI)**			**100.0%**	**0.85 [0.80, 0.91]**
Heterogeneity: Tau² = 0.01; Chi² = 44.98, df = 12 (P < 0.0001); I² = 73%				
Test for overall effect: Z = 4.95 (P < 0.00001)				

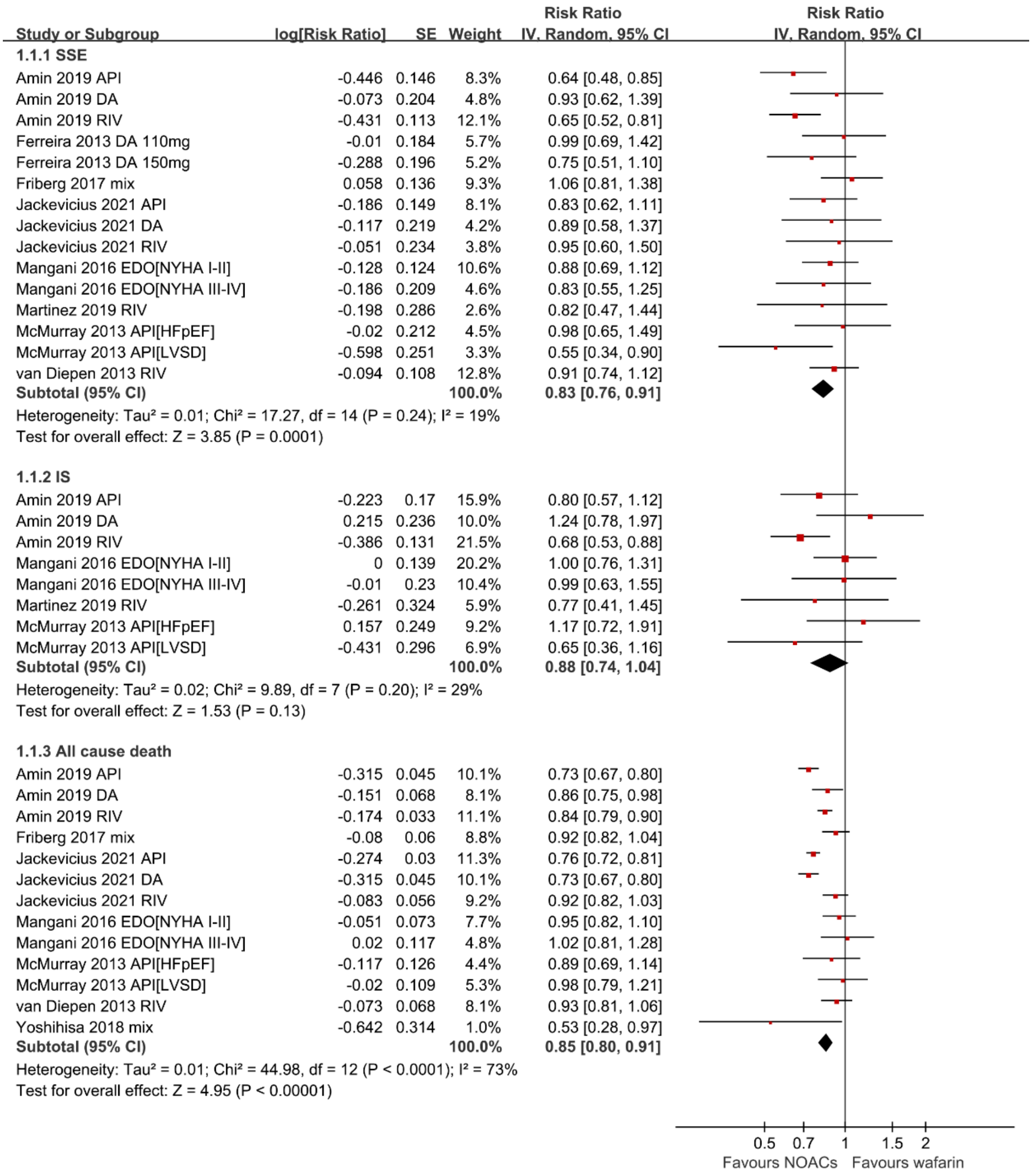

Supplementary Figure 1. Forest plot for comparing the effectiveness outcomes of NOACs with warfarin in patients with AF and HF.

Abbreviations: AF, atrial fibrillation; HF, heart failure; SSE, stroke or systemic embolism; IS, ischemic stroke; NYHA, New York Heart Associ-ation; LVSD, left ventricular systemic dysfunction; HFpEF, heart failure with reduced preserved ejection fraction; NOACs, non-vitamin K antagonist oral anticoagulants; DA, dabigatran; RIV, rivaroxaban; API, apixaban; CI, confidence interval.

Study or Subgroup	log[Risk Ratio]	SE	Weight	Risk Ratio IV, Random, 95% CI
**1.2.1 MB**				
Adeboyeje 2017 API	-0.616	0.2	4.7%	0.54 [0.36, 0.80]
Adeboyeje 2017 DA	-0.163	0.09	6.9%	0.85 [0.71, 1.01]
Adeboyeje 2017 RIV	0.104	0.094	6.8%	1.11 [0.92, 1.33]
Amin 2019 API	-0.416	0.069	7.2%	0.66 [0.58, 0.76]
Amin 2019 DA	-0.117	0.1	6.7%	0.89 [0.73, 1.08]
Amin 2019 RIV	0.166	0.047	7.5%	1.18 [1.08, 1.29]
Ferreira 2013 DA 110mg	-0.186	0.136	6.0%	0.83 [0.64, 1.08]
Ferreira 2013 DA 150mg	-0.236	0.138	5.9%	0.79 [0.60, 1.04]
Friberg 2017 mix	-0.01	0.113	6.4%	0.99 [0.79, 1.24]
Jackevicius 2021 API	-0.693	0.198	4.7%	0.50 [0.34, 0.74]
Jackevicius 2021 DA	-0.693	0.198	4.7%	0.50 [0.34, 0.74]
Jackevicius 2021 RIV	-0.494	0.194	4.8%	0.61 [0.42, 0.89]
Mangani 2016 EDO[NYHA I-II]	-0.236	0.099	6.7%	0.79 [0.65, 0.96]
Mangani 2016 EDO[NYHA III-IV]	-0.236	0.197	4.8%	0.79 [0.54, 1.16]
Martinez 2019 RIV	-0.02	0.149	5.7%	0.98 [0.73, 1.31]
McMurray 2013 API[HFpEF]	-0.478	0.177	5.1%	0.62 [0.44, 0.88]
McMurray 2013 API[LVSD]	-0.211	0.172	5.2%	0.81 [0.58, 1.13]
**Subtotal (95% CI)**			**100.0%**	**0.79 [0.69, 0.90]**
Heterogeneity: Tau² = 0.06; Chi² = 96.32, df = 16 (P < 0.00001); I² = 83%				
Test for overall effect: Z = 3.45 (P = 0.0006)				
**1.2.2 ICH**				
Amin 2019 API	-0.598	0.19	14.9%	0.55 [0.38, 0.80]
Amin 2019 DA	-0.673	0.275	7.6%	0.51 [0.30, 0.87]
Amin 2019 RIV	-0.58	0.148	22.9%	0.56 [0.42, 0.75]
Ferreira 2013 DA 110mg	-1.079	0.445	3.0%	0.34 [0.14, 0.81]
Ferreira 2013 DA 150mg	-0.942	0.422	3.3%	0.39 [0.17, 0.89]
Jackevicius 2021 API	-0.494	0.227	10.8%	0.61 [0.39, 0.95]
Jackevicius 2021 DA	-0.02	0.372	4.2%	0.98 [0.47, 2.03]
Jackevicius 2021 RIV	-0.186	0.325	5.5%	0.83 [0.44, 1.57]
Mangani 2016 EDO[NYHA I-II]	-0.799	0.244	9.4%	0.45 [0.28, 0.73]
Mangani 2016 EDO[NYHA III-IV]	-1.05	0.469	2.7%	0.35 [0.14, 0.88]
Martinez 2019 RIV	-0.315	0.54	2.0%	0.73 [0.25, 2.10]
McMurray 2013 API[HFpEF]	-1.609	0.539	2.1%	0.20 [0.07, 0.58]
McMurray 2013 API[LVSD]	-1.386	0.574	1.8%	0.25 [0.08, 0.77]
van Diepen 2013 RIV	-0.462	0.239	9.8%	0.63 [0.39, 1.01]
**Subtotal (95% CI)**			**100.0%**	**0.54 [0.46, 0.63]**
Heterogeneity: Tau² = 0.00; Chi² = 13.73, df = 13 (P = 0.39); I² = 5%				
Test for overall effect: Z = 7.89 (P < 0.00001)				
**1.2.3 GIB**				
Amin 2019 API	-0.4	0.099	12.1%	0.67 [0.55, 0.81]
Amin 2019 DA	0.058	0.138	11.4%	1.06 [0.81, 1.39]
Amin 2019 RIV	0.451	0.066	12.5%	1.57 [1.38, 1.79]
Jackevicius 2021 API	-0.198	0.201	10.1%	0.82 [0.55, 1.22]
Jackevicius 2021 DA	-0.329	0.249	9.1%	0.72 [0.44, 1.17]
Jackevicius 2021 RIV	-0.236	0.204	10.0%	0.79 [0.53, 1.18]
Mangani 2016 EDO[NYHA I-II]	0.199	0.143	11.3%	1.22 [0.92, 1.61]
Mangani 2016 EDO[NYHA III-IV]	0.068	0.284	8.3%	1.07 [0.61, 1.87]
McMurray 2013 API[HFpEF]	0.708	0.326	7.5%	2.03 [1.07, 3.85]
McMurray 2013 API[LVSD]	-0.301	0.312	7.7%	0.74 [0.40, 1.36]
**Subtotal (95% CI)**			**100.0%**	**1.00 [0.76, 1.31]**
Heterogeneity: Tau² = 0.15; Chi² = 67.15, df = 9 (P < 0.00001); I² = 87%				
Test for overall effect: Z = 0.01 (P = 0.99)				

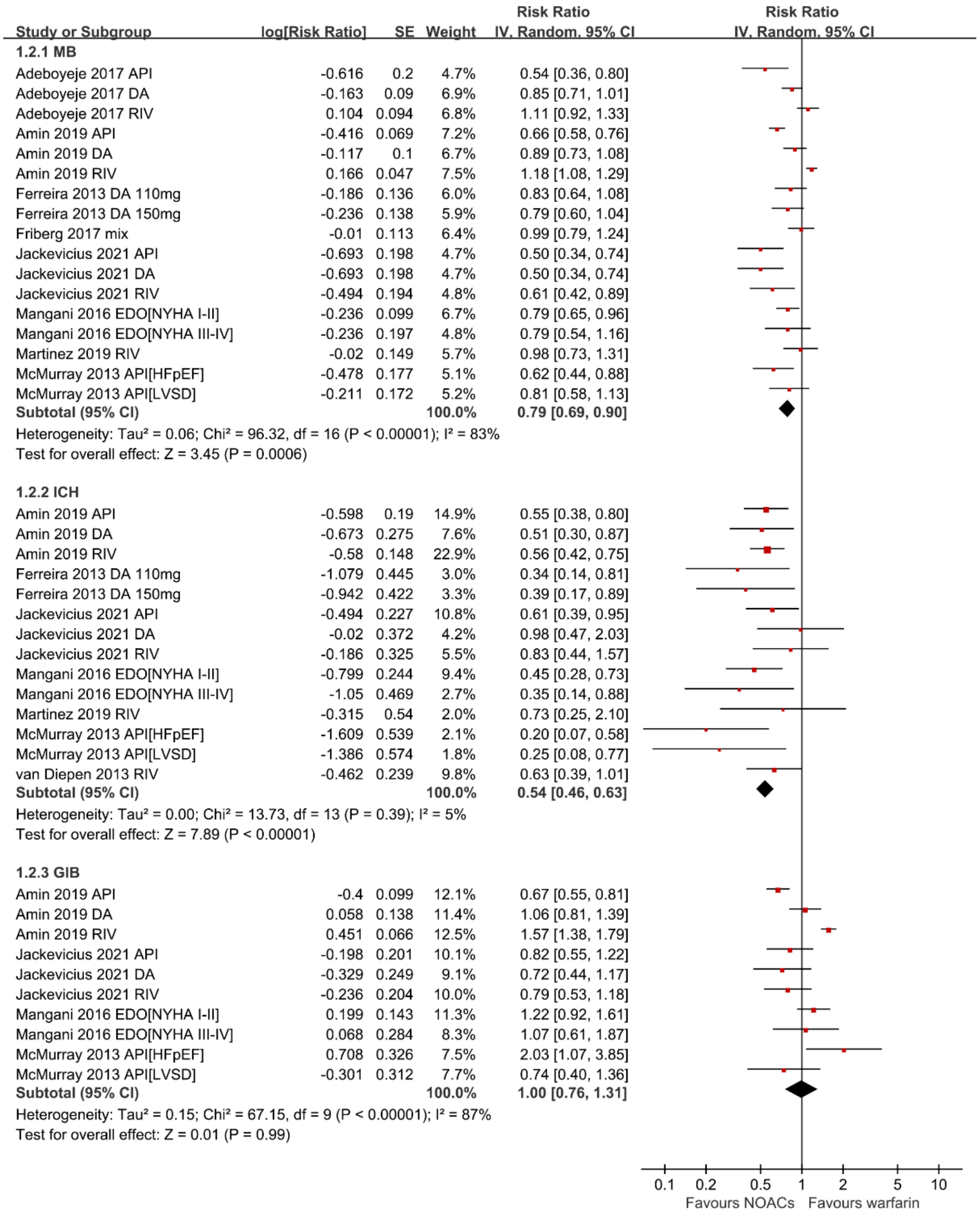

Supplementary Figure 2. Forest plot for comparing the safety outcomes of NOACs with warfarin in patients with AF and HF.

Abbreviations: AF, atrial fibrillation; HF, heart failure;MB, major bleeding; ICH, intracranial hemorrhage; GIB, gastrointestinal bleeding; NYHA, New York Heart Associ-ation; LVSD, left ventricular systemic dysfunction; HFpEF, heart failure with reduced preserved ejection fraction; NOACs, non-vitamin K antagonist oral anticoagulants; DA, dabigatran; RIV, rivaroxaban; API, apixaban; CI, confidence interval.

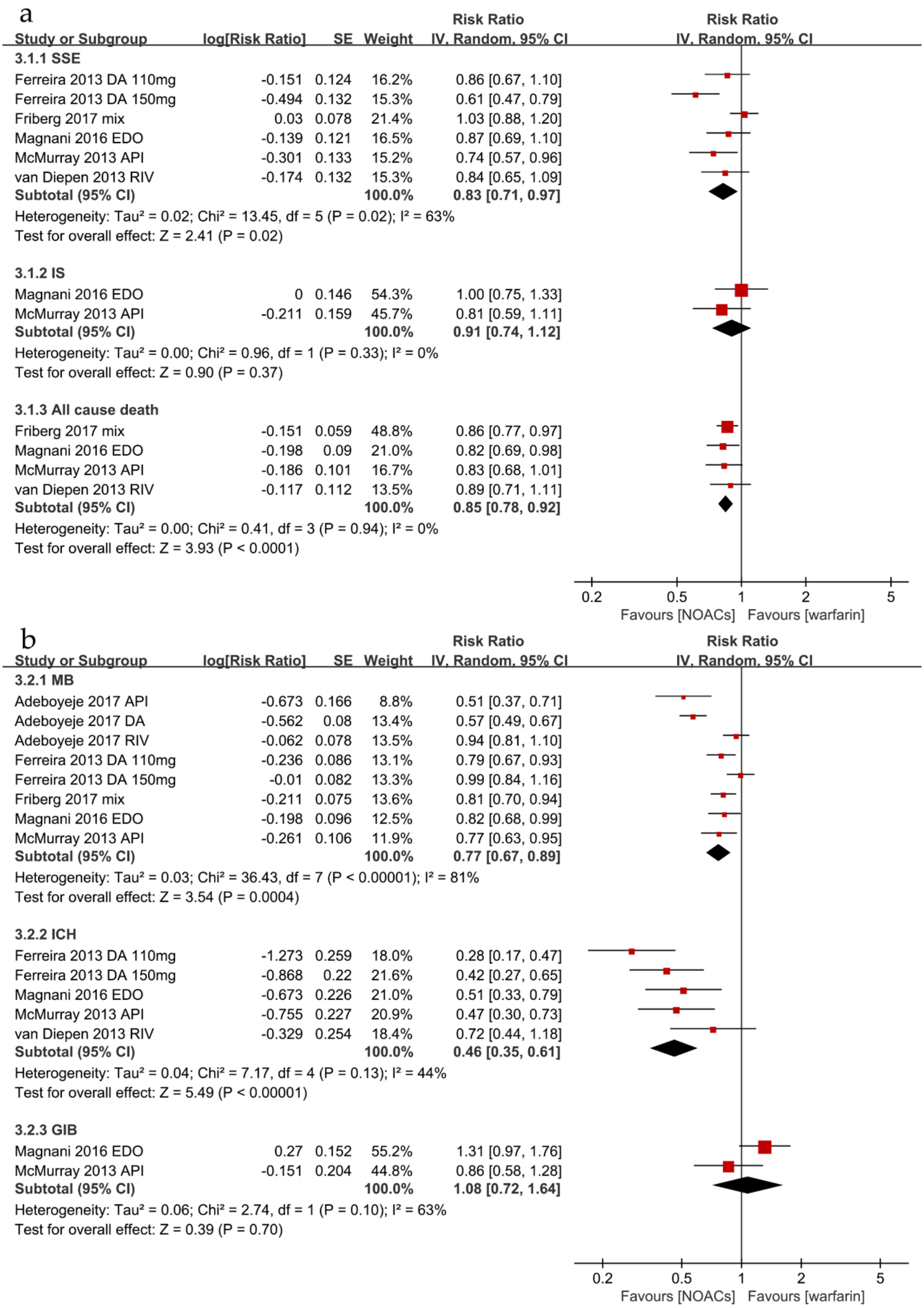

Supplementary Figure 3. Forest plot for comparing the effectiveness (a) and safety (b) outcomes of NOACs versus warfarin in patients AF without HF. Abbreviations: AF, atrial fibrillation; HF, heart failure; SSE, stroke or systemic embolism; IS, ischemic stroke; MB, major bleeding; ICH, intracranial hemorrhage; GIB, gastrointestinal bleeding; NYHA, New York Heart Associ-ation; LVSD, left ventricular systemic dysfunction; HFpEF, heart failure with reduced preserved ejection fraction; NOACs, non-vitamin K antagonist oral anticoagulants; DA, dabigatran; RIV, rivaroxaban; API, apixaban; CI, confidence interval.

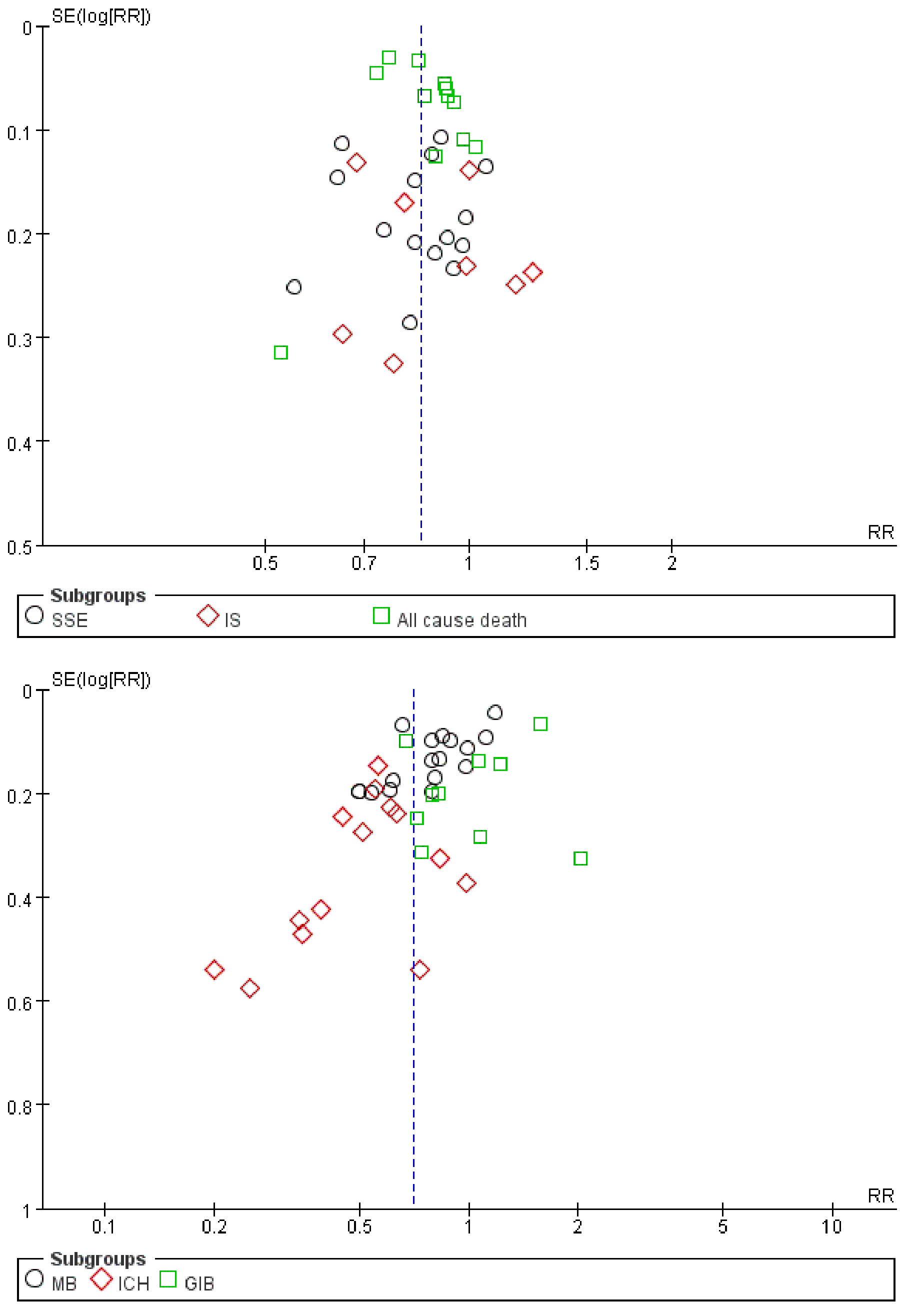

Supplementary Figure 4. The funnel plot of the reported outcomes of NOACs versus warfarin in the AF and HF.

SSE, Stroke or systemic embolism; IS, Ischemic stroke; MB, Major bleeding; ICH, Intracranial bleeding; GIB, Gastrointestinal bleeding;

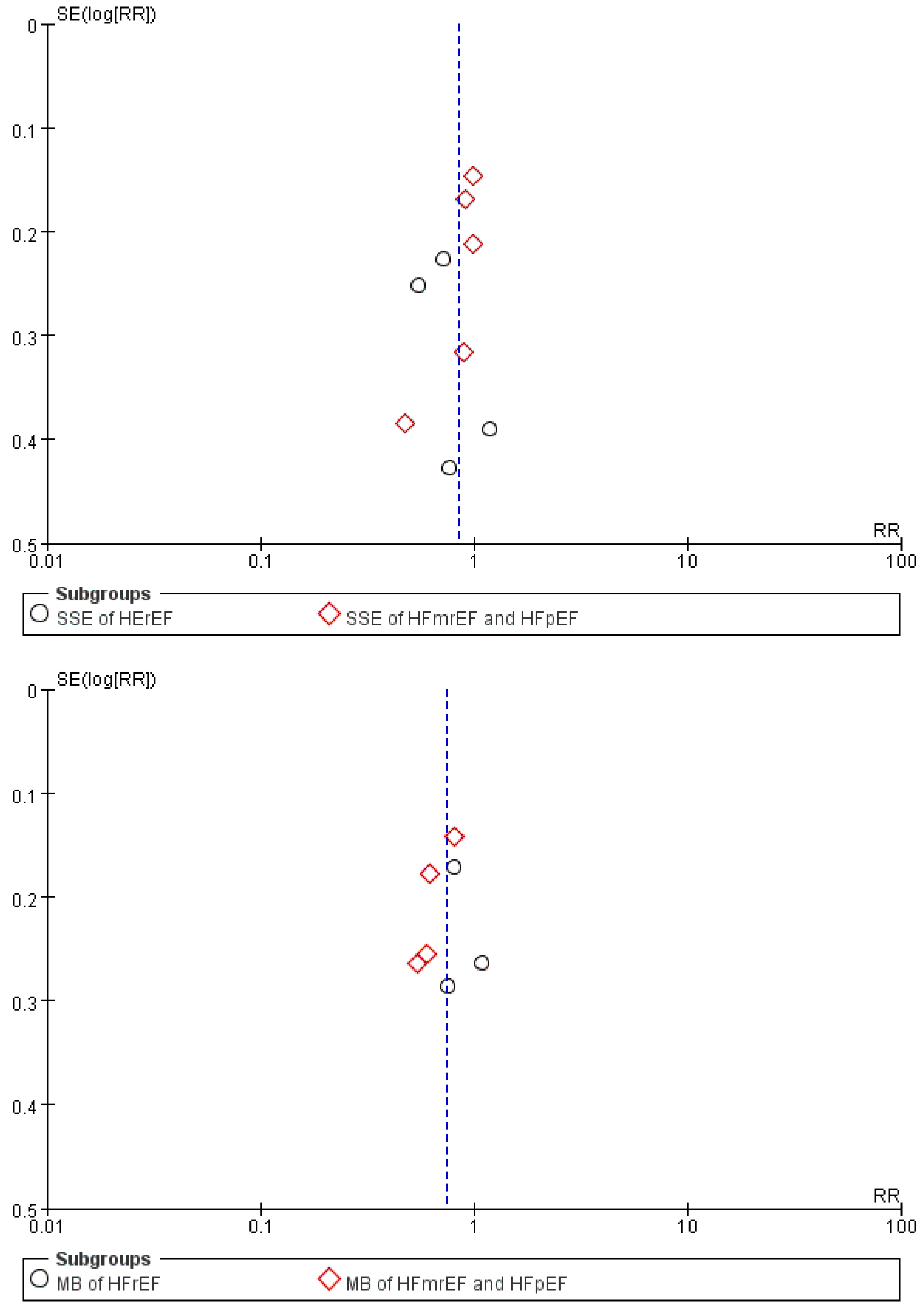

Supplemental Figure 5. The funnel plot for reported outcomes of NOACs versus warfarin in the AF and HFrEF, HFmrEF and HFpEF groups.
SSE, Stroke or systemic embolism; IS, Ischemic stroke; MB, Major bleeding; ICH, Intracranial bleeding; GIB, Gastrointestinal bleeding;
